# Reading Food Experiences from the Face: Effects of Familiarity and Branding of Soy Sauce on Facial Expressions and Video-Based RPPG Heart Rate

**DOI:** 10.3390/foods10061345

**Published:** 2021-06-10

**Authors:** Rene A. de Wijk, Shota Ushiama, Meeke Ummels, Patrick Zimmerman, Daisuke Kaneko, Monique H. Vingerhoeds

**Affiliations:** 1 Wageningen Food & Biobased Research Institute, Wageningen University & Research, 6700 AA Wageningen, The Netherlands; s.ushiama@kikkoman.nl (S.U.); meeke.ummels@wur.nl (M.U.); monique.vingerhoeds@wur.nl (M.H.V.); 2 Kikkoman Europe R&D Laboratory B.V., 6709 PA Wageningen, The Netherlands; d.kaneko@kikkoman.nl; 3 Noldus Information Technology, 6709 PA Wageningen, The Netherlands; Patrick.Zimmerman@noldus.nl

**Keywords:** RPPG and PPG heart rate, facial expressions, emojis, branding, familiarity, soy sauce

## Abstract

Food experiences are not only driven by the food’s intrinsic properties, such as its taste, texture, and aroma, but also by extrinsic properties such as visual brand information and the consumers’ previous experiences with the foods. Recent developments in automated facial expression analysis and heart rate detection based on skin color changes (remote photoplethysmography or RPPG) allow for the monitoring of food experiences based on video images of the face. RPPG offers the possibility of large-scale non-laboratory and web-based testing of food products. In this study, results from the video-based analysis were compared to the more conventional tests (scores of valence and arousal using Emojis and photoplethysmography heart rate (PPG)). Forty participants with varying degrees of familiarity with soy sauce were presented with samples of rice and three commercial soy sauces with and without brand information. The results showed that (1) liking and arousal were affected primarily by the specific tastes, but not by branding and familiarity. In contrast, facial expressions were affected by branding and familiarity, and to a lesser degree by specific tastes. (2) RPPG heart rate and PPG both showed effects of branding and familiarity. However, RPPG heart rate needs further development because it underestimated the heart rate compared to PPG and was less sensitive to changes over time and with activity (viewing of brand information and tasting). In conclusion, this study suggests that recording of facial expressions and heart rates may no longer be limited to laboratories but can be done remotely using video images, which offers opportunities for large-scale testing in consumer science.

## 1. Introduction

Food perception typically starts before the food is placed in the mouth. Before the food is consumed, consumers typically smell the food and see the food, often with the food package with brand name. Consequently, prior to tasting, consumers will already have expectations regarding the food’s taste and flavor based on visual, smell, tactile, and sometimes even auditory cues. These expectations affect whether the consumer will eat the food. Numerous studies have focused especially on visual cues. They have demonstrated the effects of branding, names and sensory descriptors, and health and ingredient labels on food choice decisions. These “extrinsic” variables provide consumers with information regarding the product’s quality, which is part of “brand equity” [1,2]. Brand equity combines perceived quality with factors such as brand loyalty, name awareness, and other associations that the consumer may have with the brand [3]. Obviously, the factors vary with the consumer’s experience with the product and aid consumers in selecting products that fit their attitudes [4,5]. Extrinsic variables may also exert direct effects on taste experiences [6,7].

Models such as the assimilation and contrast model [6,8] describe various ways of how expectations interact with actual experiences, depending on their overlap and discrepancies. In the assimilation model, the discrepancy is relatively small, and experiences are adjusted to the expectations. In contrast, when the discrepancy is relatively large, the differences between expectations and experiences are amplified. As a result, ratings for the experience will shift in the opposite direction of the expectations. Recently, examples of assimilation and contrast were provided by Głuchowski et al. [9], who investigated emotional and hedonic reactions to traditional and new innovative food dishes. The results showed that for traditional dishes, expected liking based on visual aspects was in line with experienced liking during tasting, whereas for new innovative dishes the experienced liking fell short compared to the expected liking. The results also demonstrate the importance of taking consumers’ characteristics such as food neophobia and consumer innovativeness into account.

Most studies used subjective ratings and questionnaires, so-called explicit measures, to assess the effects of expectations on taste experiences, i.e., these studies rely on introspection while consumers may not even be aware of the way expectations affect their experiences (e.g., [10]). Additionally, virtually all studies measured the effect of expectation indirectly by measuring its effect on subsequent taste experiences, i.e., only very few studies try to measure the expectations themselves, except for Thomson, who uses a specially developed technique [11].

On the other hand, implicit measures of autonomic nervous system (ANS) responses, such as heart rate, and their behavioral correlates, such as facial expressions, offer a promising alternative. In fact, these measurements (1) do not rely on introspection and offer insight into the subconscious as well as conscious processes and (2) can be measured continuously during expectations and experiences (e.g., [12]). The continuous measurement of ANS responses and facial expressions facilitate insights in the different stages of the temporal development of responses and have been corner stones of theories of emotions, such as appraisal theories. Early responses reflect aspects of stimuli, such as the stimulus’ novelty, relevance, or pleasantness. Later responses include cognitive reactions on the earlier responses.

Despite their potential, facial expressions and ANS responses have been used only in relative few food studies and with varying success: some studies failed to find effects on ANS responses or facial expressions between the tastes of foods, such as chocolates [13], beers [14], and energy drinks [15], whereas others found significant effects, even though they were small and difficult to interpret (e.g., [16] for juices, [17] for beers, and [18] for breakfast drinks). In studies where the foods were not only tasted but also visually inspected, the results typically showed clearer differences between foods. In a recent study, Bercik et al. [19] measured facial expressions as well as eye movements for three dishes with the same ingredients but served in three different ways. Results showed that the way the food was visually presented had significant emotional effects and influenced the way participants visually inspected the foods. Similarly, facial expressions were intensified when foods were tested in a natural consumption environment, such as one’s own home, rather than in the unrealistic and uninspiring setting of a sensory laboratory [20], which suggests that this type of measure reflects the complete food experience determined by a combination of variables such as the food, the physical location, and the social environment, rather than by specific taste experiences.

Unfortunately, ANS measurements and some facial expression measurements typically cannot be used in realistic non-laboratory settings because they require sensors attached to face and body [9,13,14,16,18,21,22,23,24]. Recent technical developments have resulted in video-based automated analysis of facial expressions and even more recently in video-based recordings of heart rate using remote photoplethysmography or RPPG (e.g., [20,25]). If these new methods prove to be a good alternative for the gold-standard methods such as electrocardiogram (ECG) and photoplethysmography (PPG), then these measurements would no longer require physical contact between the sensor and human volunteer. This would open a whole new range of applications such as testing in realistic consumption environments, and internet testing using a webcam, which is built-in in most notebooks nowadays.

This study explored the use of video-based facial expressions, RPPG heart rate, and Emoji scores of valence and arousal [26], for branded and unbranded foods. The results of this study complement results from the same study reported previously. Those results, based on invasive PPG heart rates, as well as skin conductance measures, showed that facial expressions, PPG heart rate, and skin conductance varied significantly with the level of familiarity of product for the consumer and with the type of branding and varied less with the brand of soy sauce. Visual analogue scale (VAS) liking and arousal scores also varied with the level of familiarity and with the brand of soy sauce. In contrast with the other measures, VAS scores did not vary with branding [27].

This study aims at (1) the comparison of valence and arousal from video-based facial expressions with Emoji scores during food tasting, and (2) the comparison of video-based RPPG heart rate and PPG heart rate during food viewing and tasting. Specifically, the sensitivity of the methods to detecting effects of branding, soy sauce, and degree of consumers’ familiarity will be compared. For these purposes, samples of branded and unbranded soy sauces are presented to experienced and inexperienced consumers. During visual inspection and tasting, video-based facial expressions, RPPG and PPG heart rate, and Emoji scores of valence and arousal [26] were recorded.

## 2. Materials and Methods

### 2.1. Participants

Forty healthy participants (30 females and 10 males aged between 20 and 59 yrs. with an average age of 43 yrs.) were recruited from the consumer database of Food, Health, and Consumer Research (Wageningen Food and Biobased Research) after screening with an online survey (EyeQuestion Software, Logic8 B.V., The Netherlands). The database consists of 2945 persons (1015 males and 1930 females all living in or near Wageningen and aged between 19 and 95 yrs). Exclusion criteria were allergies or intolerance to wheat, gluten, soybean, or rice allergy. All participants provided written consent to participate in the experiment. In the recruitment questionnaire, participants were asked to indicate the frequency of their soy sauce use using an 8-point scale (more than three times a week, 1–2 times a week, once a month, once per 3–4 month, 1–2 times a year, less than once a year, never used, and I do not know if I use soy sauce) and daily use soy sauce brand. The participants were assigned to one of two groups depending on their frequencies of use. Twenty-two participants, 17 females and 5 males, who used soy sauce either never or maximum once per month were assigned to the low-frequency user group. Eighteen participants, 15 females and 3 males, who used soy sauce at least once per week were assigned to the high-frequency user group.

The participants received financial compensation of 10 euro after finalizing the study. The study was approved by the Social Ethical Committee of Wageningen University and Research.

### 2.2. Soy Sauces

Three soy sauces were selected based on the similarity of ingredients and availability in the Netherlands. Soy sauce is a condiment with a distinctive flavor that is familiar to most Asian consumers, and nowadays, a large variety of soy sauces are increasingly being offered in regular supermarkets to the mainstream consumers. Despite its growing popularity, soy sauce is still consumed regularly by a relatively small group of consumers, while most consumers either never consume soy sauce or very infrequently [28]. Recently, the frequency of use of soy sauce was related to factors such as food neophobia and preferences for soy sauces in general as well as preferences for specific brands of soy sauce [29]. Based on this study, two familiar brands of soy sauces (Kikkoman (Kikkoman Foods Europe B.V., Sappemeer, The Netherlands) and Inproba (Inproba B.V., Baarn, The Netherlands)) were selected that shared the typical savory salty taste of soy sauce and one unfamiliar brand of soy sauce with a clearly different taste (Maekrua Gold Label^®^ Soya Sauce Formula 1 (Mae Krua Sri Ruen Company Limited, Bangkok, Thailand)). In order to study the effect of brand familiarity, the familiar Inproba soy sauce was presented in an unfamiliar bottle (Wan Ja Shan from Taiwan). Samples of the soy sauces (1 mL, either with or without branding information) were mixed together with 16 g of shaped Sticky-rice (Yumenishiki, JFC Deutschland GmBH, Düsseldorf, Germany) in a styrofoam cup and presented at a comfortable temperature. White rice with soy sauce was selected as a carrier because it is a relatively common way to use soy sauce in the Netherlands. Rice had been cooked in a commercial rice cooker and kept warm at 60 °C in the incubator and portioned before each session.

### 2.3. Procedure

Participants participated in one 1-h session, which started with an explanation of the procedure, followed by the attachment of the PPG heart rate sensor on the fingers of the non-dominant hand, and by two practice trials. Participants were seated in front of a laptop with a webcam (Microsoft LifeCam) that recorded their face. Instructions and images of the unbranded or branded soy sauces were shown on the laptop screen using E-Prime software (E-Prime, Science Plus Group, Groningen, The Netherlands). Participants were instructed that their perception of soy sauces was studied, and that to avoid unnecessary movements that may interfere with the measurements, small samples of rice with soy sauce would be put in their mouth by the experimenter. The sample should be chewed normally, and the participant was instructed to signal the swallow with a hand signal, after which scores were entered by a mouse click.

**Scheme 1:**
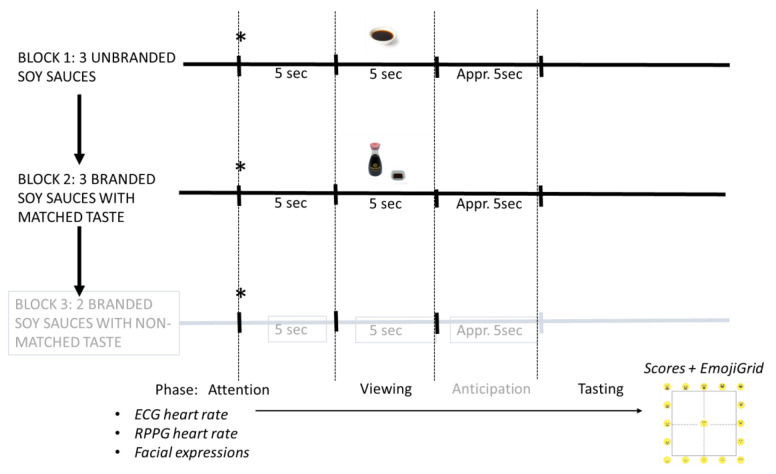
Schematic representation of the study procedure. Participants were presented with 3 blocks of trials. Each trial started with an attention signal (asterisk), followed by an image of branded or unbranded image soy sauce, a viewing/anticipation period, and a period where rice with soy sauce was chewed. ECG and RPPG heart rate, skin conductance, and facial expressions were recorded continuously. VAS and EmojiGrid scores were recorded at the end of each trial. Heart rates, facial expressions, and Emoji scores for block 1 and 2 are reported in this manuscript.

**Figure 1:**
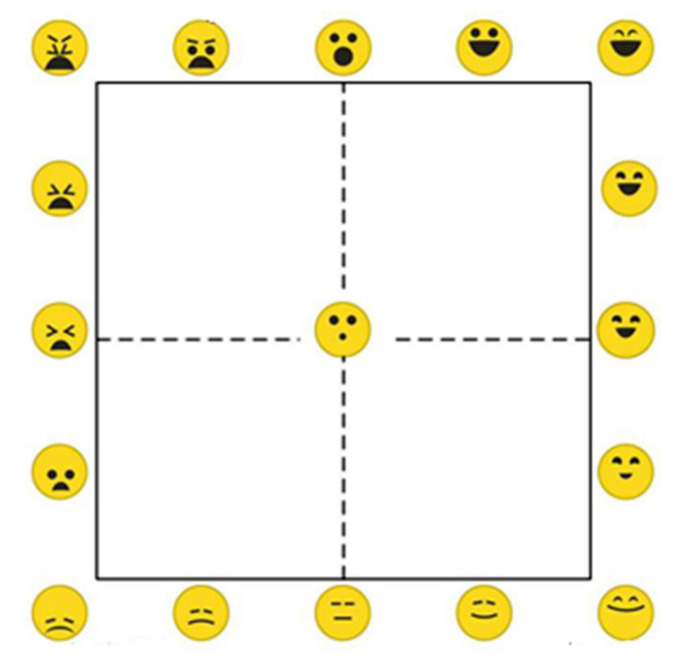
The EmojiGrid used in the study to measure valence and arousal [25].

Next, participants received three blocks of stimulus trials in which he/she received taste samples of cooked rice mixed one of three soy sauces. In each trial, participants, either familiar or unfamiliar with soy sauces, first received an auditory warning signal followed after 5 s by an image (*viewing phase*) for 5 s showing either a generic volume of soy sauce in a white cup (unbranded block 1), or the same white cup with soy sauce combined with the bottle of one of the soy sauces showing the package of the product (blocks 2 and 3, with matched branding and non-matched branding, respectively). After the 5 s viewing period, the image disappeared during an anticipatory waiting period of approximately 5 s. Next, the image reappeared and the participants were given a spoon of the mixture of rice and soy sauce in the mouth by the experimenter who simultaneously pressed the enter key of the keyboard to signal the start of tasting (*tasting phase*) in the data recordings. In the branded match block 2, the brand shown in the image and the tasted soy sauce were the same, whereas they were different in the branded non-match block 3 (results will not be reported in here). After each sample, which was chewed typically for at least 10 s, the participant first scored the liking of the sample using a visual analogue scale (VAS), followed by a just-right scale for saltines. Next, participants indicated the perceived valence and arousal using a so-called “EmojiGrid” [26,30] (see Figure 1). Only the Emoji scores of valence and arousal will be reported here. Heart rate responses and facial expressions were recorded during each of the phases in all trials. Each of the three soy sauces was presented twice in block 1 and four times in block 2 in a randomized order. To avoid possible unwanted interference of the non-matched condition with the other conditions, the non-matched condition (not reported here) was tested last in block 3 with only two of the three soy sauces presented once. This resulted in a total number of 20 trials presented at a rate of approximately 1 trial per 150 s. The order of the blocks was the same for all participants. The procedures are shown schematically in Scheme 1.

### 2.4. Physiological ANS Measures

PPG heart rate was collected using a BIOPAC MP150 system and AcqKnowledge 5.0.4 data acquisition software (BIOPAC Systems Inc., Santa Barbara, CA, USA). Heart rate was measured using the TSD200 PPG transducer attached to the index finger of the non-dominant hand. The TSD200 transducer was also connected to the PPG/EDA transmitter. Data were stored on a desktop PC (sample rate 250 Hz). The PPG signal was processed real-time in AcqKnowledge using the Pulse Rate calculation channel, resulting in the pulse/heart rate in beats-per-minute (bpm).

RPPG heart rate was recorded remotely based on photoplethysmography (RPPG), a technique that measures the small changes in color caused by changes in blood volume under the skin epidermis using a multi-wavelength RGB camera [31,32]. Pilot food studies in which RPPG heart rate was compared to the gold-standard ECG and PPG during chewing and not-chewing showed that RPPG heart rate was generally approximately 6 BPM lower than ECG and PPG heart rate, especially during chewing. Overall, the correspondence between RPPG and ECG/PPG heart rate was satisfactory [33].

Heart rate in combination with other physiological parameters have previously been related to food preferences and emotions (see [12]).

### 2.5. Facial Expressions

Video segments of the consumption of each bite were stored together with the participant’s code, the product code, and time and date information. Facial expression data were automatically analyzed per time frame of 0.04 s by FaceReader 8.0 (Noldus Information Technology, Wageningen, The Netherlands) in three steps. The face is detected in the first step using the Viola-Jones algorithm [34]. Next, the face is accurately modelled using an algorithmic approach [35]. Based on the Active Appearance method described by Cootes and Taylor [36], the model is trained with a database of annotated images that describes over 500 key points in the face and the facial texture of the face. Finally, the actual classification of the facial expressions is based on an artificial neural network trained with 10,000 manually annotated images. The face classification provides the output of seven basic expressions (happy, sad, angry, surprised, scared, disgusted, and contemptuous), one neutral state on the basis of the Facial Action Coding System developed by Ekman and Friesen [37], three “affective attitudes” (interest, boredom, and confusion), and arousal and valence dimensions based on combinations of facial expressions. Valence scores were calculated per time frame from the FaceReader happiness score minus the most intense FaceReader negative emotion score (sad, angry, surprised, scared, disgusted, and contemptuous). FaceReader scores for each emotional expression, except arousal, range from 0 (emotion is not detected) to 1 (maximal detection) and are based on intensity judgments of human experts. Arousal scores range from −1 to 1. FaceReader allows for the simultaneous presence of multiple emotions. Only the arousal and valence dimensions were used for this study.

FaceReader was validated by others using the Radboud Faces Database, a standardized test with images of expressions associated with basic emotions. The test persons in the images were trained to pose a particular emotion and the images were labelled accordingly by the researchers. Subsequently, the images were analyzed in FaceReader. Accuracy of the assessment of the emotions by FaceReader varied between 84.4% for scared and 95.9% for happy, with an average of 90% [38]. Other validation studies showed superior performance of FaceReader for neutral faces (90% correct recognition for FaceReader versus 59% for humans) [39].

A more detailed description of the science behind FaceReader can be found at http://info.noldus.com/free-white-paper-on-facereader-methodology/ (accessed on 18 May 2021). Whether all possible emotional expressions can be categorized by these six emotions, affective attitudes, and arousal/valence dimensions remains a matter of debate (e.g., [40]).

### 2.6. Data Analysis

*Physiological responses and facial expressions.* Facial expressions and heart rates were collected during the five-second intervals of the viewing, anticipation, and tasting phases. The duration of the anticipation phase was not exactly controlled because it ended when the participant was manually fed the test food and started chewing, and only the results of the viewing and tasting phases will be reported here. For each phase, averages per second were used for further analysis. PPG heart rate was not successfully collected for five participants. To allow the proper comparison, RPPG heart rates of these participants were also omitted from further analysis. For the remaining participants, heart rates were missing in 4% of all cases, and facial expressions were missing in 1% of all cases.

*EmojiGrid scores.* The positions of the scores on the EmojiGrid valence (X) and arousal (Y) axis were converted into numerical values (1–10).

Results were analyzed with Mixed Model Anovas (IBM^®^ SPSS^®^ statistics, version 25, Armonk, New York, NY, USA) with the participant as a random factor, and with the familiarity with soy sauce (familiar and unfamiliar users), soy sauce (3), duration (5 s), and condition (unbranded and branded) as fixed factors. The usage of soy sauce was a between-subject factor, and the others were within-subject factors. LSD post-hoc tests were used for main effects. 95% Confidence intervals were used for post-hoc tests of interaction effects.

## 3. Results

### 3.1. General

The results of facial expressions and RPPG/PPG heart rates will be presented for the viewing and tasting phases, together with Emoji valence and arousal scores, which were collected only in the tasting phase. The effects of each phase will be presented, as well as the effects of soy sauce, familiarity with soy sauce, and branding (no branding, matched branding). Post-hoc test results will only be reported when they are significant.

### 3.2. Viewing Phase: Facial Expressions

*Facial expressions of valence*. Facial expressions to images of foods became more negatively valenced when branding information was added (F(1,1092) = 27.2, *p* < 0.001). The effects of branding varied with the type of soy sauce (interaction F(2,1092) = 3.9, *p* = 0.02). Facial expressions did not vary with time.

**Figure 2:**
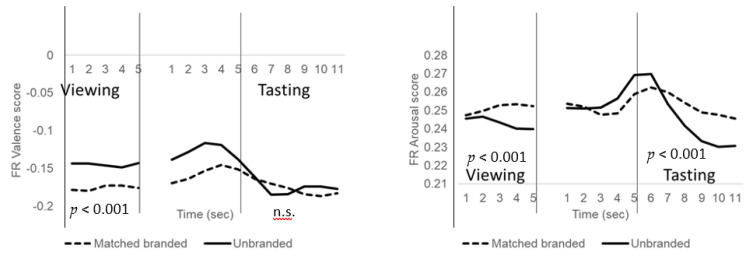
Facial expressions related to valence (left) and arousal (right) during 5 s of viewing, 5 s of anticipation between viewing and tasting, and 6 s of tasting (6–11 s) of unbranded (solid line) and matched branded (dashed line) stimuli. Results were averaged across participants and stimuli. Significance levels of branded/unbranded differences are indicated by *p*-values. N.s. = not significant.

*Facial expressions of arousal*. Facial expressions related to arousal became intensified with branding information (F(1,1092) = 35.4, *p* < 0.001). Facial expressions varied overall between soy sauces (F(2, 1091) = 3.4, *p* = 0.03), and varied with combination of soy sauce and experience (interaction: F(2,1091) = 4.5, *p* = 0.01). Branding affected familiar and unfamiliar participants differently (interaction: F(1,1091) = 4.3, *p* = 0.04). Facial expressions did not vary with time. Results are shown in Figure 2.

### 3.3. Viewing Phase: RPPG and ECG Heart Rate

*RPPG heart rate.* RPPG heart rates during viewing of the food lowered from 59.7 to 57.9 BPM when branding information was also presented (F(1,1080) = 24.0, *p* < 0.001). RPPG heart rates varied between soy sauces (F(2,1080) = 3.2, *p* = 0.04), and with combinations of soy sauce, familiarity, and branding (F(2,1080) = 5.5, *p* < 0.01). RPPG heart rate did not vary with time.

**Figure 3:**
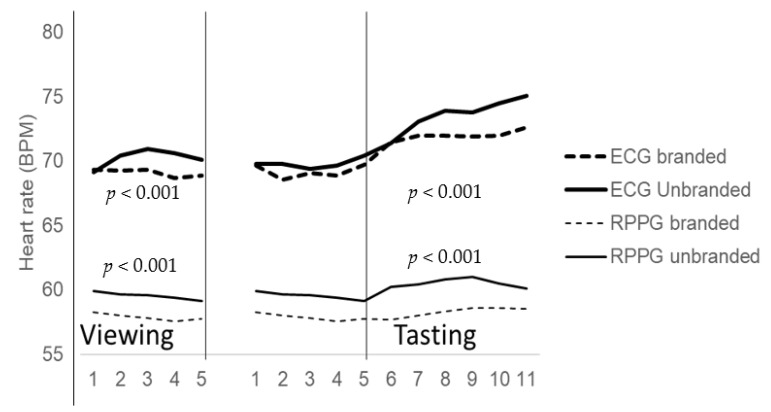
Heart rates measured by ECG and RPPG during 5 s of viewing, 5 s of anticipation between viewing and tasting, and 6 s of tasting (6–11 s) of unbranded (solid line) and matched branded (dashed line) stimuli. Significance levels of branded/unbranded differences are indicated by *p*-values.

*PPG heart rate.* PPG heart rates during viewing were 9–10 BPM higher than RPPG heart rates but showed a similar decrease with branding information (from 70.6 to 68.6 BPM, F(1,957) = 78.6, *p* < 0.001). The effect of branding varied with the participants’ familiarity (F(1,957) = 10.2, *p* = 0.001). The lowering of PPG heart rate with branding information was larger for unfamiliar participants. PPG heart rate did not vary with time. Results are shown in Figure 3.

### 3.4. Tasting Phase: Facial Expressions

*Facial expressions of valence*. Facial expressions became increasing more negatively valenced during tasting (effect of time: F(4,1099) = 7.4, *p* < 0.001). Brand information was associated with more negatively valenced facial expressions in low-frequency users, whereas branding did not affect facial expressions of high-frequency users (interaction: F(1,1099) = 5.8, *p* = 0.02).

*Facial expressions of arousal*. Overall, arousal was more intense at the beginning of tasting and became less intense there after (F(4,1098) = 27.9, *p* < 0.001). Facial expressions during tasting became more aroused with branding information (F(1,1098) = 27.2, *p* < 0.001). The branding effect was strongest for inexperienced participants (F(1,1098) = 5.5, *p* = 0.02). Expressions of experienced participants were most aroused by Kikkoman, whereas those of inexperienced participants were most aroused by Inproba (interaction F(2,1098) = 3.3, *p* = 0.04). Results are shown in Figure 2.

### 3.5. Tasting Phase: RPPG and PPG Heart Rate

*RPPG heart rate*. RPPG heart rates during tasting lowered from 60.2 to 57.8 BPM with branding information (F(1,1074) = 44.5, *p* < 0.001). This decrease was especially large for inexperienced participants (interaction: F(1,1074) = 7.6, *p* < 0.001). RPPG heart rates of experienced participants were highest for Kikkoman, whereas those for inexperienced participants were highest for Formula 1 (interaction: F(2,1073) = 13.7, *p* < 0.001).

*PPG heart rate*. PPG heart rates during tasting were 12–14 BPM higher than RPPG heart rates, but also showed a lowered PPG heart rate (from 74.6 to 71.9 BPM) with branding information (F(1,954) = 116.2, *p* < 0.001). PPG heart rates varied with soy sauce (F(2,954) = 5.2, *p* < 0.001). PPG heart rates increased during chewing from 71.6 to 74.6 BPM (F(4,954) = 17.2, *p* < 0.001). This increase was especially large for inexperienced participants (F(4,954) = 2.8, *p* = 0.02). Results are shown in Figure 3.

### 3.6. Explicit Valence and Arousal during Tasting

Emoji liking (EmojiX scores) varied significantly with soy sauce (F(2,190) = 6.2, *p* < 0.01). Kikkoman was liked best (6.1), followed by Formula1 (5.7) and Inproba (5.3). Inclusion of (matched) branding information or familiarity group did not affect Emoji liking scores. Emoji arousal (EmojiX scores) also varied significantly with soy sauce F(1,190) = 4.5, *p* = 0.01). Arousal was strongest for Kikkoman (3.2) and Formula1 (3.2) and weakest for Inproba (2.70). Inclusion of (matched) branding information or familiarity group did not affect Emoji arousal scores.

## 4. Discussion

The study investigated the effects of consumer’ experience and branding on reactions to the sight and taste of branded and unbranded soy sauces. This study aimed at (1) the comparison of valence and arousal from video-based facial expressions and from Emoji scores during food tasting, and (2) the comparison of video based RPPG heart rate and PPG heart rate during food viewing and tasting.

With regard to the first aim, the study showed differences between the results from implicit (facial expressions and heart rate) and explicit (scores) responses. Explicit Emoji taste scores of arousal and valence showed significant differences between the brands of soy sauce but showed no effects of branding and consumer’ experience. In general, facial expressions of arousal and valence primarily showed effects of branding and consumer’ experiences. Moreover, these differences were not only found during tasting but also prior to tasting when the participants’ looked at the unbranded and branded foods. This illustrates the importance of consumers’ expectations based on so-called extrinsic food factors, such as the food’s visible appearance, the packaging, and branding information. It also suggests that measures such as facial expressions and heart rate are better suited to investigate the role of extrinsic factors than the more traditional measures such as scores. Overall, this outcome corresponds with other results of the same study reported earlier [27], as well as results of a recent study that also showed that scores reflected differences between brands of soy sauce but were not affected by branding [29].

The PPG and facial expression results from the same study reported previously [27] failed to show systematic differences between soy sauces, whereas in the results reported here occasional differences between soy sauces were found during viewing as well as tasting. These differences are probably related to differences in the way the results of the earlier and this study were analyzed. In the previous study, changes in heart rate, and facial expressions, relative to the start of viewing and tasting were analyzed, whereas in this study absolute values were used. The fact that differences between soy sauces were found in the absolute values and not in the relative values suggest that these differences do not develop during tasting but have already developed previously during the viewing of the food and branding information, and the following anticipation phases, and are continued during tasting. This illustrates how measures such as facial expressions and heart rate provide a window into processes that start prior to tasting with expectations based on visual (as well as possibly olfactory and auditory) information and that affect experiences during subsequent tasting. These interactions between expectations and actual taste experiences are not (always) reflected in scores. The richness of the processing during visual inspection and subsequent tasting that is reflected in the heart rate and facial expressions may also be a reason why heart rates, and responses of the autonomic system in general, do not always show differences between products when they are based solely on taste responses. For example, Gunaratne [41] presented participants with unbranded chocolate samples and failed to find effects of specific chocolate tastes on heart rate and skin temperature (see also [13,14], who showed similar results for chocolate and beer, respectively). Other studies did demonstrate significant product effects on ANS responses, but these effects were typically small and difficult to interpret (e.g., [18]). The higher sensitivity of heart rate and facial expressions compared to explicit scores for factors such as branding that are not directly related to the food’s taste properties is in line with our previous study. In that study, participants evaluated foods repeatedly in their own home and in the sensory laboratory [20]. Factors such as time of the day, day of the week, and sample preparation and presentation procedure were kept constant, whereas the physical location was systematically varied. Again, the results showed that explicit scores reflected primarily the differences between the test foods, whereas the heart rate and facial expressions reflected differences between test locations as well. Combined, the results of these studies suggest that physiological measures and facial expressions may reflect experiences in general and not only experiences directly related to foods. These experiences may be related to a specific mood that is affected by the food that is consumed and the circumstances in which the food is consumed. A close link between the consumption context and how consumers feel has been postulated by others [42], and these feelings are thought to be underlying modulators of food perception, food liking, and overall enjoyment of human eating experiences [43]. Others, such as Meiselman [44], have also stressed the importance of assessing foods and drinks in accurate contexts in order to increase the external validity of the results of consumer tests. The present study demonstrates that physiological measures and facial expressions may be especially well suited for the assessment of general food experiences.

Subjective rating scores such as the Emoji scores used in this study are single point measurements where all the consumer’ processes during viewing, anticipation, and tasting are collapsed over time into one single score. An obvious result is that many of the nuances pre- and during tasting are blunted and possibly lost. Facial expressions and heart rate offer a continuous measurement of the pre- and during tasting processes. The fact that facial expressions and PPG heart rate vary between viewing and tasting, and show variation over time, illustrates that these consumer’ processes are highly dynamical. In order to understand consumer’ processes, these dynamics need to be considered when selecting the appropriate measurement. This means that the measurements need to be continuous, like facial expressions and heart rate, and not static like scores.

The second aim of the study was the comparison of heart rate recorded with non-invasive video-based RPPG and with invasive PPG (which relates closely to ECG, the ground truth). A successful comparison would mean that physiological heart rate recordings are no longer limited to laboratory studies but could be also applied to studies in realistic consumer situations. This is important because consumer’ reactions to foods are not only driven by food properties but also by the physical and social context in which the food is consumed. Another possible new application would be internet-based surveys where large numbers of consumers can be reached. Unfortunately, the comparison of RPPG and PPG heart rates indicates that at this moment the RPPG method cannot fully replace the PPG and other invasive methods. RPPG heart rates were systematically approximately 10–12 BPM slower than PPG heart rates, and this difference seemed to be larger for higher PPG heart rates associated with for example chewing activity. Similar results were reported previously by Kanemura et al. [33], who speculated that some of the differences could be related to the effects of chewing activity on skin color. In addition, PPG heart rates changed rapidly over time during viewing and tasting, whereas RPPG heart rates were relatively stable. Rapid changes in PPG heart rates have been associated with orienting responses to new and unexpected stimuli and attentional processes (e.g., [21,45]). The results of this study suggest that the current RPPG heart rate measurements cannot (yet) be used to study this detailed level of processes. Even though RPPG heart rates based on video images currently lack the high level of sensitivity provided by “gold-standard” measurements, RPPG heart rates showed overall effects of consumers’ experience, phase (viewing, anticipation, and tasting) and occasionally of brand of soy sauce, similar to PPG heart rates. Obviously, further development of the RPPG technique is needed for a closer match with PPG heart rates, and additional research is needed to explore the relation between both types of heart rate measurements further.

This research contributes—together with other studies—to the understanding of how experiences and expectations from packaging and branding interact with subsequent taste experiences. The results clearly demonstrate that taste reactions are directly related to reactions to packages and branding, and that both need to be considered for the development of new products. In traditional sensory research, taste experiences are typically tested with unbranded products, i.e., without any packaging and branding information. In subsequent consumer tests, only products that successfully pass the initial sensory taste test were tested together with branding and packaging information. This, as well as other research, indicate that this step-wise approach may not be the most efficient way to develop new successful products (see also [46]). This study also demonstrates the need to not only assess effects of branding/packaging on taste reactions but also to measure reactions to the branding/packaging itself. There reactions are especially difficult for consumers to articulate because they are often unaware of them. In those cases, measures such as facial expressions and physiological measurements may provide important insights, not only into the effects of branding/packaging but also into the interactions with specific consumer characteristics, such as the consumers’ experience with the brand or product.

In conclusion, this study suggests that video-based measurements of facial expressions and RPPG heart rates offer opportunities for large-scale web-studies to investigate consumers’ reactions to extrinsic food factors such as packages, labels, logos, and product information. In addition, facial expressions and RPPG heart rates may be well suited to study the effects of consumers’ traits, such as level of experience with the foods, on their reactions to extrinsic food factors.

## Data Availability

The data presented in this study are available on request from the corresponding author.
